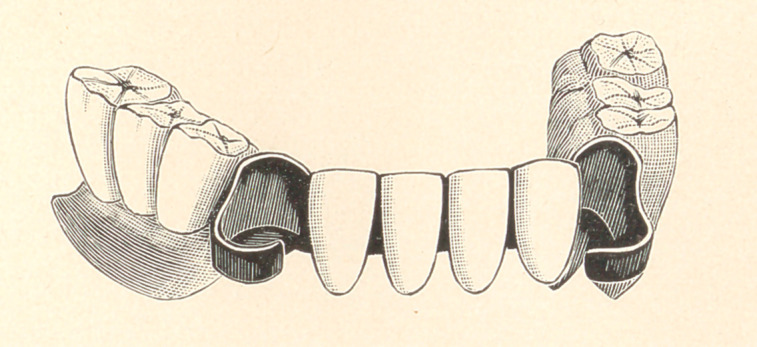# Crown- and Bridge-Work

**Published:** 1892-09

**Authors:** C. M. Richmond

**Affiliations:** New York


### CROWN- AND BRIDGE-WORK.[1]

BY DR. C. M. RICHMOND, NEW YORK.

(Continued from page 578.)

IN my August article I have shown a crown (detachable). I did not give the full details of its construction, as that will come in the papers on crowns.

I have taken for my third article two cases of plate and bridge combination, both of which are nearly alike in appearance, although the methods of construction are different. As I have both cuts, I illustrate them.

The first case is a lower piece with five teeth in front, one incisor being lost. It will be seen by reference to Fig. 1 that the gold attachments are made to fit the teeth perfectly, without in the least impinging upon the gum tissues. The first step in the construction of this case is to take an impression of the cuspid teeth with plaster, and by placing a thin piece of steel or other thin metal between the tooth to be taken and the adjoining tooth, I can get a perfect impression with the two teeth apart, so that a fusible metal die can be cast into the impression of the cuspid alone, and thus secure the means of fitting a perfect clasp to the two eye-teeth, as shown in Fig. 2.

After having perfect models of these teeth, they are fitted with a band of twenty-two-carat gold, thirty in [illegible]ickness (United States standard guage), entirely to the gum line, letting the bands meet, and soldering them together at the side where the opening is shown in Fig. 1. I then fit around this band a band of clasp metal. The first band is now invested inside only, and I leave enough of the investment to serve as a base for it to rest on and remain upright while soldering. After the two bands are invested in this way, a clasp-band is slipped over them, and, after heating to the proper degree, small pieces of solder are dropped onto the edges of these bands and the two gradually soldered together, and by lowering the

---

[1] Copyrighted, 1892, by Dr. C. M. Richmond.

Model of plaster.

FIG. 1.

FIG. 2.

Teeth on model.

FIG. 3.

clasp-band a little lower than the gold one room is secured to begin to solder. A solid clasp is very easily made with a soft and perfectly-fitting gold surface next to the teeth. I cut them apart at the sides next to the adjoining teeth, as shown in Fig. 2. After the clasps are finished and fitted to the two canines, a tooth is waxed (to fill in the space where the one is lost) into position by drying the surfaces of the natural teeth, and also covering the facial surface of the porcelain with hard wax, and thus a tooth is firmly held long enough to take an impression of the lingual surfaces for the purpose of casting a fusible metal die.

When this is done a perfect surface is secured over which to burnish pure gold between the clasps, which I now have in a model in fac-simile. After this piece of pure gold has been perfectly fitted by burnishing, it is invested, leaving the surface I wish to solder exposed. This must now be covered with clasp-metal and solder. I make with my clasp-benders pieces of clasp-metal to fit each tooth-surface which has been brought out in the pure gold burnishing, and by laying these into position on the gold the solder is applied, and gradually the pure gold and clasps are all soldered together in one piece, and I have here also a plate with a soft piece of gold to fit the tooth-surface, while the strength is accomplished with the clasp-gold soldered on to the surfaces, as in the bands I have already described.

Having the clasp and the plate finished, they must now be fitted together in the mouth and soldered in one piece, and therefore the bands are placed onto the canine teeth, and while in position the plate is fitted between these bands, so that it can be tied into proper position on the teeth with a piece of silk.

After all are in position, a plaster impression is taken of the case, and upon removal thereof I take off my bands and plate and fit them into position in the impression, and into this I pour investment. After this is hard the plaster is cut away and the case is put into the fire, and the plate and the two clasps are soldered together in one piece; this is now put onto the teeth in the mouth, and, if all is correct, a perfect-fitting piece of work is produced that will go down to its place on the teeth and no farther, and it is immovable except when taken off by the hand of the patient. This part of the work, being well done, is the groundwork for the whole case. To attach the bicuspids and molars is an easy operation, and is done by placing the part finished in position and taking a plaster impression of the mouth back of the plate; then, by placing the plate into the impression, a plaster model is prepared and a plaster fac-simile of the case is produced, with the parts that are finished in position.

A pattern of tin is now made the size I wish to make my plate, and I then cut the plate (which is pure gold, thirty in thickness). This can be put onto the plaster model and carefully worked down to its place, and it can be made to fit perfectly in this way. This is then waxed to the clasps and the gold, and it can be carefully removed from the model and the two gold plates soldered to the clasp, after having been invested. It is now replaced on the model, and if the plates fit the model perfectly when the front piece is in position it is ready for the articulation, which is done in the ordinary way, and the teeth are ground into position. Gum teeth were used in this case and banded to finish the outside, while solder made the inside finish, as the front tooth is placed in position by drilling two holes through the gold which crosses the space where the front tooth is gone and is waxed with hard wax, and being a tooth with long pins it is easily held where it belongs until the last investment, where all the teeth are soldered to the work.

In the case (Fig. 3) here presented is a bridge between the canines, and it was worn for weeks before the molars and bicuspids were put on the construction, and this is accomplished by making the two canine bands like the previous ones, and then having the incisor teeth backed up in the usual way. They are waxed into position and tried in the mouth, two teeth being waxed to each band, and when in proper position a plaster impression is taken, and, after placing the two bands and the four teeth into the impression, investment is poured over it. After having cut away the plaster impression, the teeth are soldered together and attached to the bands. The bridge part is now finished. This case can now be taken off and replaced as many times as one may require to adjust the back teeth, which are backed up in the usual way and waxed onto the case, one at a time, until they are perfectly articulated with the upper set of teeth, and then they are all soldered to the bands, and are at the same time made fast together, one with the other, so that there is now a set of teeth held in position by two bands on the cuspidati, no part of the work touching the gums.

I wish to put a small saddle of pink rubber under the molars and bicuspids, so that there will be no motion to the structure while in use. This is accomplished by making a small base of wax on the lower ends of these teeth, to serve as an impression-cup. Plaster is now mixed, and the wax is filled with enough to take an impression of the gums directly under the molars and bicuspids.

After the case is removed a model is poured into the impression, and the teeth are now in position on a plaster cast, showing the space where pink rubber is to be placed as a base to support the work from the canines back. The cast is waxed up and put into the vulcanizer, leaving the teeth and gold part exposed, so that when the flask is separated the gold and teeth will come out in the upper half of the flask, and leave only the model of the lower gum in the lower half. Pink rubber is now packed and the flask put together in the usual way, and the case is vulcanized, as is shown in Fig. 3. I take a disk and groove the lower end of these teeth so that the pink rubber will have a perfect surface to fasten to. This case, like the first one, does not impinge on the gum tissues in the least degree at the canines or between them, and is what I choose to term plate and bridge combinations.

The first case is worn by Dr. A. L. Northrop, and the latter one by Dr. W. H. Dwinelle.

I am giving only practical cases, and shall confine my articles to such as have been in successful use for a period of time to prove them practicable. I refer to this subject because, while Dr. Farrar was publishing his articles on regulating, I frequently heard men high in our profession say, "That looks well on paper; I wonder if it will prove successful." "It is all theory," etc., while the fact is, every case published by Dr. Farrar was a practical one, and the models are at this writing in his office, where they can be seen and admired by all who will take the trouble to look at them.

(To be continued.)

## Figures and Tables

**Fig. 1. f1:**
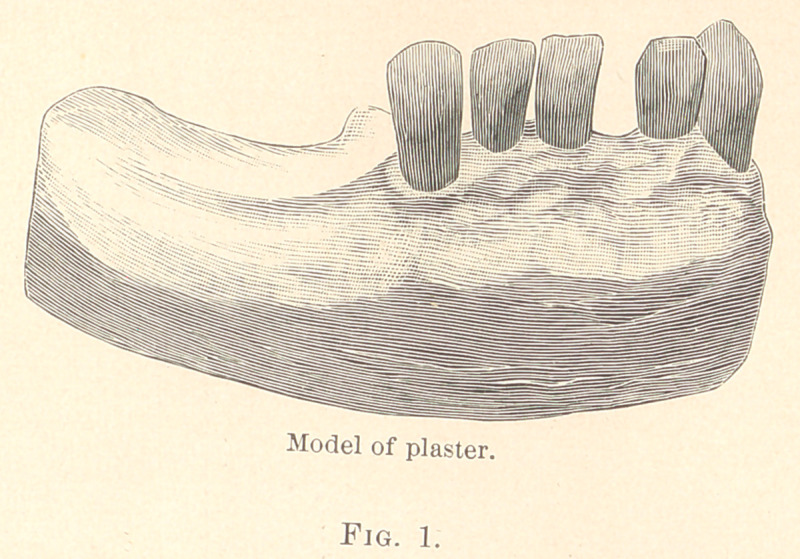


**Fig. 2. f2:**
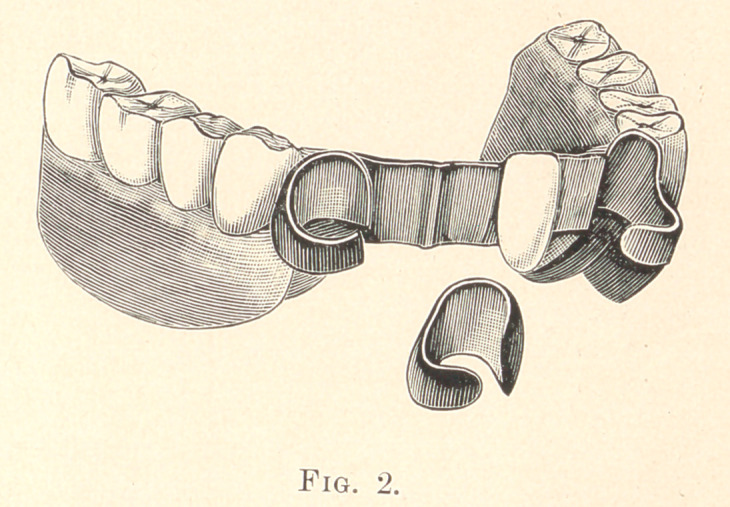


**Fig. 3. f3:**
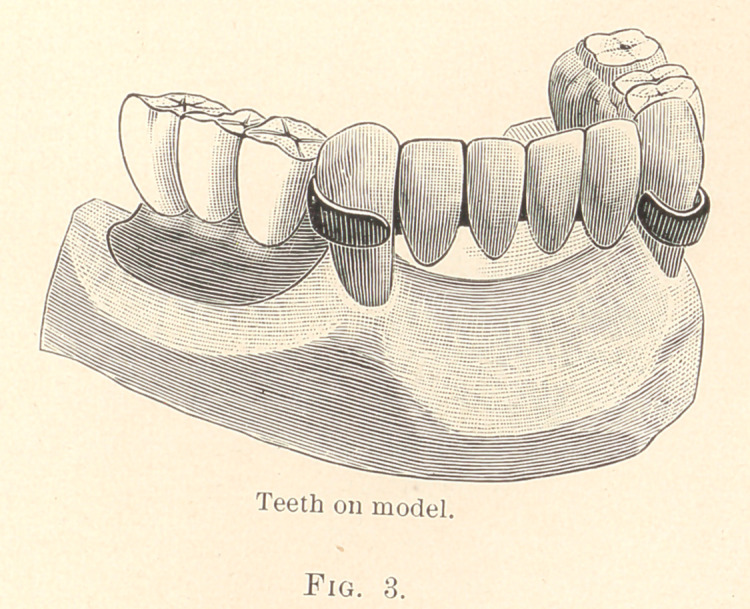


**Figure f4:**